# Cross-species epigenetic regulation of nucleus accumbens KCNN3 transcripts by excessive ethanol drinking

**DOI:** 10.21203/rs.3.rs-3315122/v1

**Published:** 2023-09-15

**Authors:** Rita Cervera Juanes, Patrick Mulholland, Audrey Padula, Larry Wilhelm, Byung Park, Kathleen Grant, Betsy Ferguson

**Affiliations:** Atrium Health Wake Forest Baptist; MUSC; Oregon Health & Science University; Oregon Health & Sciences University/Oregon National Primate Research Center

## Abstract

The underlying genetic and epigenetic mechanisms driving functional adaptations in neuronal excitability and excessive alcohol intake are poorly understood. Small-conductance Ca^2+^-activated K^+^ (K_Ca_2 or SK) channels encoded by the *KCNN* family of genes have emerged from preclinical studies as a key contributor to alcohol-induced functional neuroadaptations in alcohol-drinking monkeys and alcohol dependent mice. Here, this cross-species analysis focused on *KCNN3* DNA methylation, gene expression, and single nucleotide polymorphisms including alternative promoters in *KCNN3* that could influence surface trafficking and function of K_Ca_2 channels. Bisulfite sequencing analysis of the nucleus accumbens tissue from alcohol-drinking monkeys and alcohol dependent mice revealed a differentially methylated region in exon 1A of *KCNN3* that overlaps with a predicted promoter sequence. The hypermethylation of *KCNN3* in the accumbens paralleled an increase in expression of alternative transcripts that encode apamin-insensitive and dominant-negative K_Ca_2 channel isoforms. A polymorphic repeat in macaque *KCNN3* encoded by exon 1 did not correlate with alcohol drinking. At the protein level, K_Ca_2.3 channel expression in the accumbens was significantly reduced in very heavy drinking monkeys. Together, our cross-species findings on epigenetic dysregulation of *KCNN3* represent a complex mechanism that utilizes alternative promoters to impact firing of accumbens neurons. Thus, these results provide support for hypermethylation of *KCNN3* as a possible key molecular mechanism underlying harmful alcohol intake and alcohol use disorder.

## Introduction

Alcohol (ethanol) use disorder (AUD) is a devastating brain disease driven by complex interactions between genetic, epigenetic, and environmental factors. Genetic factors increase the propensity for risky drinking^1, 2^, chronic ethanol intake, are associated with neuroepigenetic alterations^3–8^, and influences the efficacy of treatment options for individuals with AUD^9^. In addition, prolonged excessive ethanol consumption produces neuroadaptations in projection neurons and neural circuits that are proposed to sustain heavy drinking^10–12^. Although there are three FDA-approved pharmacotherapies for treating AUD, only one (i.e., naltrexone) targets genetic variation in individuals with AUD. While pharmacologically targeting known single nucleotide polymorphisms (SNPs) can reduce relapse rates in a subpopulation of individuals with AUD^9, 13^, mixed results suggest a critical need for further investigation of the (epi)genetic factors and neuroadaptations that contribute to the excessive ethanol drinking in AUD.

Small-conductance Ca^2+^-activated K^+^ (K_Ca_2 or SK) channels in cortical-striatal brain circuitry involved in motivational processing have emerged from preclinical studies as a target for treating AUD^10, 14–19^. In this circuitry, neuronal firing patterns^20^ of nucleus accumbens core (NAcC) medium spiny neurons (MSNs) and substantia nigra dopamine neurons are controlled by K_Ca_2.3 channels encoded by the *KCNN3*. Previous functional mouse genomics studies, found quantitative trait loci (QTL) containing *Kcnn3*, particularly NAcC *Kcnn3* transcript levels, were negatively correlated with voluntary drinking in genetically diverse BXD strains^17, 18^. Further, the promoter region of *Kcnn3* was associated with ethanol preference in selectively bred rat lines^21^. In addition, intrinsic excitability and reduced K_Ca_2 channel function and K_Ca_2.3 channel protein expression were increased in the NAcC of excessive ethanol drinking and dependent rodents^15, 17, 22^. Functionally, blocking K_Ca_2 channels in the NAcC with apamin increased voluntary ethanol drinking in mice^17^, whereas positive modulators of K_Ca_2 channel function reduced home cage drinking and operant self-administration^15, 19, 22^. Importantly, the ability of apamin to inhibit K_Ca_2 channel function was completely lost in NAcC MSNs from ethanol dependent mice, but not in rats that had access to 7-weeks of operant self-administration of moderate amounts of ethanol^15^. Together, these studies identified *KCNN3* in general, and NAcC K_Ca_2 channel function in particular, as a potential regulator of excessive ethanol consumption and dependence in rodent models of chronic ethanol exposure.

There are two polymorphic CAG repeats in the N-terminus of human *KCNN3* encoded by exon 1^23^. Higher numbers of CAG repeats reduced K_Ca_2 channel function in transfected HEK293 cells^24^, and this polymorphism has been associated with neuropsychiatric conditions, such as schizophrenia and anorexia nervosa^23, 25–28^. While this polymorphism did not confer risk for developing the disease, longer CAG repeat length is associated with higher cognitive performance in individuals with schizophrenia^24^. This finding is consistent with a known role for K_Ca_2.3 channel regulation of cognitive function and plasticity of intrinsic excitability that is an important mechanism for forming new learned associations ^24, 29, 30^. Because the CAG trinucleotide repeat is conserved in nonhuman primates^24, 29–31^ and macaques exhibit a range of ethanol drinking that mimics human consumption^32–34^, the present study explored the relationship between CAG repeat length and ethanol drinking in rhesus macaques with low- and heavy-drinking phenotypes.

DNA methylation (DNAm) is an epigenetic mark that contributes to modulation of gene expression by modifying the accessibility of transcription factors to chromatin. Alterations in DNAm are reported in heavy drinking humans, monkeys and rodents^3–5, 35, 36^, and a recent study demonstrated that knockdown of DNA methyltransferases reduced *Kcnn3* expression and increased intrinsic excitability of cultured cortical neurons^37^. Thus, we measured DNAm levels at a differentially methylated region in exon 1 (MR-ex1) that coincides with a cross-species regulatory region within the *KCNN3* promoter. In humans, the *KCNN3* gene encodes four known transcripts by making use of alternative first exons and alternative splicing. Similar to the CAG trinucleotide repeat, alternative *KCNN3* transcripts influence function of K_Ca_2 channels. *KCNN3* transcript *SK3_1B* encodes a truncated channel that functions as a dominant-negative to suppress endogenous K_Ca_2 channel currents^38^, whereas transcript *hSK3_ex4* encodes a protein with an additional 15 amino acid insertion within the S5-PHelix loop that renders the channel insensitive to apamin block^39^. In chronic ethanol drinking mice and monkeys, changes in DNAm of exon 1 altered the expression of *KCNN3* transcripts in the NAcC. Here, we report a complex cross-species relationship between NAcC *KCNN3* and excessive ethanol drinking that ultimately leads to reduced K_Ca_2.3 channel protein expression in mice and monkeys.

## Methods

### Ethanol self-administration in rhesus macaques

Male and female rhesus macaques (*n* = 66, *Macaca mulatta*) from seven different cohorts (cohorts 4, 5, 6a, 6b, 7a, 7b, and 10) were included in this study (**Supplemental Table 1**) and described in detail in **Supplemental Materials**. Monkeys were individually housed and ethanol self-administration was induced using schedule-induced polydipsia, as previously described^34^. For all cohorts, monkeys had open access to 4% ethanol (w/v, diluted in water) and water (ethanol subjects) or water only (control subjects) for 22 h/day, every day, for over 12 months (see^33^ for further details on these seven cohorts). The 49 monkeys with access to ethanol were classified into three different age categories based on their age of first ethanol access and four different drinking categories based on previously described criteria^32^. All of the animal procedures used in this study were approved by the Oregon National Primate Research Center IACUC and were performed in accordance with the NIH and the National Resource Council’s *Guide for the Care and Use of Laboratory Animals*.

### Ethanol dependence and two-bottle choice drinking in C57BL/6J mice

Sixty adult male C57BL/6J mice were purchased from Jackson Laboratory (Bar Harbor, ME) at ~ 7 weeks of age. Baseline ethanol drinking (22 h day/15% ethanol v/v) was established prior to treatment with 4 repeated weekly cycles of chronic intermittent ethanol (CIE) exposure in vapor inhalation chambers, alternated with weekly home cage drinking sessions, as previously described^17^ and in further detail in **Supplemental Materials**. Seventy-two h following the last vapor chamber exposure, mice were given limited access to ethanol or water for 2–3 days prior to sacrifice and tissue collection. The Medical University of South Carolina Institutional Animal Care and Use Committee approved all procedures in accordance with NIH guidelines for the humane care and use of laboratory animals.

### Genomic DNA and total RNA isolation

After the 12 month open access period, a detailed necropsy protocol was used to systematically collect tissues from all macaques^40^. Genomic DNA and RNA were extracted from male and female monkey and male mouse NAcC samples using the All Prep DNA/RNA/miRNA Universal kit (QIAGEN Sciences Inc, Germantown, MD) following the manufacturer’s recommendations. Blood samples drawn from macaques prior to ethanol self-administration were used for CAG repeat analysis. Briefly, blood was collected in EDTA tubes and DNA was isolated using QIAamp DNA mini kit following manufacturer’s instructions (QIAGEN Sciences Inc).

### Trinucleotide repeat analysis

Blood DNA was used to analyze the number of CAG repeats within the second *KCNN3* CAG repeat region as previously described^41^. The primers and methods are described in **Supplemental Materials**. We found that the first exon 1 CAG repeat was not variable in rhesus macaques; thus, these studies focused on the second exon 1 CAG repeat length.

### Bisulfite amplicon sequencing

Bisulfite amplicon sequencing was used to measure the DNAm rates of a DMR within the *KCNN3* promoter region using NAcC tissue from macaques and mice following published methods^30–31^ and as described in **Supplemental Materials**. Primers were designed to amplify a 646 bp region of the *KCNN3* within exon 1A and intron 1 in human, rhesus macaque, and mice. Because of the length of the region, two sets of primers were designed to cover the whole region (**Supplemental Table 2**).

### High-throughput real time PCR

The NAcC RNA quantity and quality was evaluated and qPCR was performed in triplicates assays, as described in **Supplemental Materials**. The primer sequences are described in **Supplemental Table 3.** Since most of the alternative transcripts are not annotated in the Rhesus or mouse genome, we used the human annotations to design the primers, then identified the homologous sequence in the rhesus macaque (MacaM)^42^ and mouse (GRCm38.p3) genome. The mRNA expression levels were normalized using the phosphoglycerate kinase (*PGK1*) gene. This gene was demonstrated to be a reliable control for brain gene expression^43^. We also previously confirmed that different levels of ethanol use did not affect its expression^4^.

### Western blot analysis

After extraction, tissue samples containing the NAcC extracted from female control and long-term drinking rhesus macaques were prepared for western blot analysis following our previously published methods in monkey brain tissue^44^, as fully described in **Supplemental Materials**.

### Statistical Analysis

Data from heavy and very heavy drinking monkeys were combined due to small sample sizes in the transcript analysis and bisulfite sequencing studies. All statistical analyses were carried out using IBM SPSS Statistics (Armonk, NY) except where noted, with values α < 0.05. The Shapiro-Wilk test (appropriate for small sample sizes) was used to assess the normality of the average methylation rate, mRNA expression rate, and K_Ca_2.3 protein expression level per comparison group. All variables analyzed followed a normal distribution. Welch’s one-way ANOVA was used to compare the difference in average methylation between controls and ethanol drinkers with Games-Howell post-hoc tests. One-way ANOVA was used to compare mRNA relative expression levels between groups. Prior to applying one-way ANOVA, Levene’s test was used to test homogeneous variance assumption for parametric methods. When heterogeneous variance was detected, we used the nonparametric Kruskal-Wallis test. Bonferroni or Tukey correction for the multiple comparisons were used to correct the overall type I error rate. Two-tailed independent t-test was used to compare the difference in average methylation rate between controls and dependent mice. Based on the Levene’s test for homogeneous variance, we used the appropriate p-value (homogeneous or heterogeneous variance). The allele frequency distribution of CAG trinucleotide repeats between controls and drinking monkeys was compared using the Kruskal-Wallis test (GraphPad Prism software, version 7.04, La Jolla, CA). Normalized western blot data were analyzed by a two-tailed t-test in Prism. Ethanol drinking data in mice were analyzed by a repeated-measures mixed linear model with a Tukey post-hoc test (SAS Institute, Cary, NC, USA).

## Results

### Ethanol drinking in monkeys and mice

**Figure 1:**
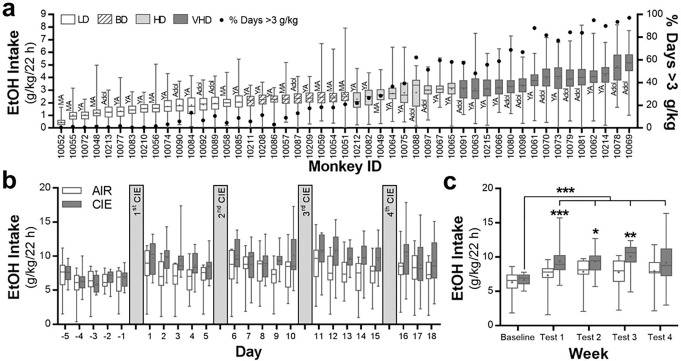
*KCNN3*-CAGnallele frequency distribution among male and female rhesus macaques. **a** The frequency distribution of (CAG)*n*alleles is shown for low drinkers (LD), binge drinkers (BD), heavy drinkers (HD), and very heavy drinkers (VHD). **b** Correlation between CAG repeat sum and average ethanol intake. **c-e** Correlations between CAG repeat sum and *KCNN3* transcript expression in long-term drinking rhesus macaques.

Sixty-six, male and female rhesus macaques, enrolled based on no common parents, were used in this study. Previously, daily ethanol drinking was mathematically modeled based and determined 4 categorical levels of intake: low, binge, heavy and very heavy. In the subjects of this study, there were 16 low drinking (LD), 9 binge drinking (BD), 9 heavy drinking (HD), and 15 very heavy drinking (VHD) monkeys. The average daily (22 h) ethanol intake (range: 0.47 to 5.15 g/kg) across the 12 months of open access for each of the drinking monkeys along with their age at drinking onset and percentage of drinking days over 3 g/kg are shown in Fig. 1a. While the adolescent and young adult monkeys occupied a range of categorical drinking, only one of the mature adult monkeys used in this study met criteria for HD or VHD. More detailed analyses of their drinking patterns have been reported previously^32, 33^.

To more closely match the monkey drinking paradigm, the standard two-bottle choice, limited-access mouse model of dependence-induced escalation of drinking was modified to allow mice open access to ethanol (15% v/v) for 22 h/day. Ethanol drinking prior to and following each weekly exposure to CIE is shown in Fig. 1b,c (n = 15 mice/group). Consistent with studies using limited access to ethanol^17, 45, 46^, mice exposed to CIE significantly increased their voluntary ethanol intake (interaction: F(4,109) = 2.47, *p* = 0.0491). Post hoc analysis indicated that the two treatment groups were not different at baseline (*p* = 0.6685), but differed during weekly test drinking sessions 1 (*p* = 0.0074), 2 (*p* = 0.0468), and 3 (*p* = 0.0034). In the ethanol dependent mice, drinking levels in all four test sessions were significantly higher than their intake during baseline (*p* = < 0.0001).

### KCNN3 polymorphisms and ethanol consumption levels

**Figure 2:**
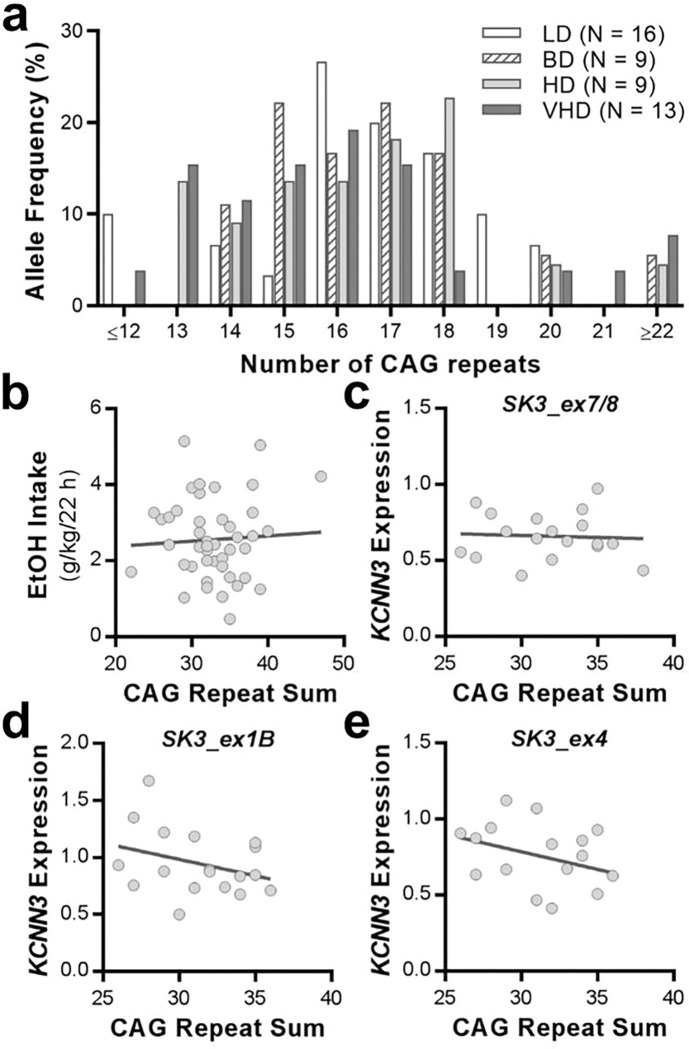
Ethanol consumption for the monkeys and mice included in the current study. **a** Average daily ethanol (4% v/v) intake in male and female rhesus macaques and their drinking category during 12 months of open access. Also shown is their age at the time of first ethanol exposure and the % days where they consumed >3 g/kg/22 h. Adol, adolescent; BD, binge drinking; HD, heavy drinking; LD, low drinking; MA, mature adult; VHD, very heavy drinking; YA, young adult. **b** Daily ethanol (15% v/v) intake values in male C57BL/6J mice prior to and after each of four cycles of chronic intermittent ethanol (CIE) exposure in vapor inhalation chambers (n = 15 mice/group). **c** Average weekly ethanol intake in mice during the week prior to and after each cycle of CIE exposure (Tukey post hoc, ***p = 0.0074 vs test 1 air group; *p = 0.0468 vs test 2 air group; **p = 0.0034 vs test 3 air group; ***p = <0.0001 tests 1–4 vs baseline CIE group).

The promoter region of MR-ex1 of *KCNN3* contains two polymorphic sites composed of a variable number of CAG repeats that have been associated with K_Ca_2 channel activity. It has been reported that higher numbers of CAG repeats reduces K_Ca_2 channel currents^24^. Thus, we investigated the variability of these polymorphisms in ethanol-drinking rhesus macaques and their influence on *KCNN3* regulation and transcript levels. In our studies, the first CAG repeat array that was not polymorphic, however the second CAG repeat copy number was highly variable across rhesus monkeys (Fig. 2a) and ranged from seven to 30 repeats. We found no differences between the frequency distributions for CAG repeats in low, binge, high and very high drinking monkeys (*H*(4) = 2.354, *p* = 0.5023; Fig. 2a). In addition, the sum of CAG repeats of both alleles did not correlate with ethanol intake values (*p* = 0.6946; Fig. 2b), the expression of KCNN3 transcripts (*p* ≥ 0.1631; Fig. 2c–e), nor averaged MR-ex1 DNAm rates (*p* = 0.3563; **Supplemental Fig. 1**). Data for expression of *KCNN3* transcripts and MR-ex1 DNAm rates were obtained from the following sets of studies.

### KCNN3 methylation analysis

**Figure 3:**
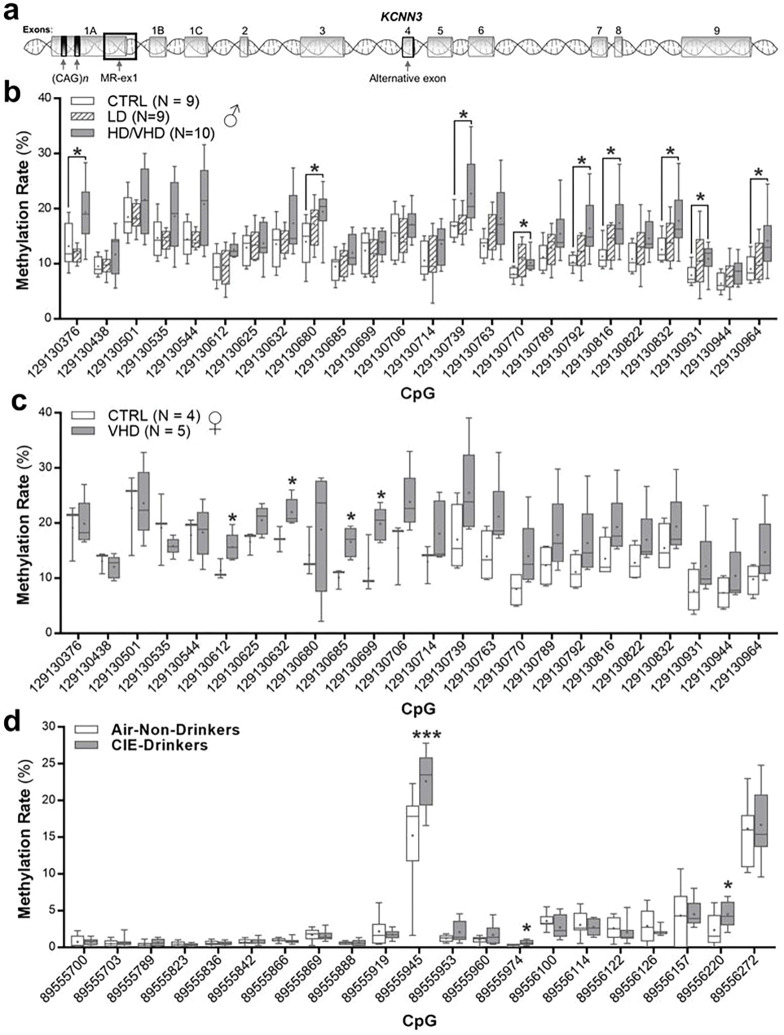
*KCNN3* methylation levels within MR-ex1-200 of ethanol drinking monkeys and dependent mice. The average methylation rates of individual CpGs included in the methylation region under study are shown. **a** Exon organization of the *KCNN3* locus showing the location of exons and the dual CAG trinucleotide repeat arrays and methylated region in exon 1 (MR-ex1). **b** In rhesus macaque, the following CpGs showed elevated rates of methylation in heavy/very heavy drinking macaques vs controls: CpG129130376: F(2, 11.846) = 7.9, *p = 0.04; CpG_129130680_: F(2, 15.955) = 3.661, *p = 0.036; CpG_129130739_: F(2, 15.468) = 3.817, *p = 0.04; CpG_129130770_: F(2, 13.57) = 5.047, *p = 0.041; CpG_129130792_: F(2, 13.945) = 7.047, *p = 0.01; CpG_129130816_: F(2, 15.63) = 5.836, *p = 0.015; CpG_129130832_: F(2, 15.473) = 3.95, *p = 0.033; CpG_129130931_: F(2, 14.393) = 4.016, *p = 0.038; CpG_129130964_: F(2, 15.708) = 3.985, *p = 0.031. **c** In female macaques, elevated rates of methylation were observed at the following CpGs in very heavy drinking macaques vs controls: CpG_129130612_: F(1, 7) = 6.122, *p = 0.048; CpG_129130632_: F(1, 7) = 7.64, *p = 0.033; CpG_129130685_: F(1, 7) = 13.370, *p = 0.011; CpG_129130699_: F(1, 7) = 7.799, *p = 0.031. **d** In mouse accumbens, the following CpGs showed elevated rates of methylation between non-drinking mice and drinking dependent mice: Independent t-tests: CpG_89555945_, *t*(16)= −2.946, ***p = 0.0009; CpG_89555974_, t(17) = −2.860, *p = 0.011; Cp_G89556220_, *t*(17) = −2.206, *p = 0.042.

By comparing the DNA sequence of the *KCNN3* gene and promoter across human, rhesus macaque, and mouse, we identified ten regions with potential conserved regulatory region, based on sharing over 95% sequence homology and ≥ 75% CpG identity. We then analyzed the DNAm patterns in these ten candidate regions between ethanol-naïve, LD, and HD/VHD rhesus macaques. Because of the small sample size, BD were not included in this analysis, while HD and VHD were combined based on their similar drinking behavior. Among the different regions, only a region of 646 bp overlapping with exon 1 and intron 1 of *KCNN3* (MR-ex1; Fig. 3a) showed significant DNAm differences between groups (Fig. 3b). The MR-ex1 region contained 24 CpGs in rhesus macaques that were 96% conserved in humans (**Supplemental Fig. 2**). While the overall CpG conservation was lower in the mouse as compared to both human and rhesus macaques (15 CpGs, 75%), the high sequence and CpG similarity of this region across species suggests functional relevance and underscores the potential translational value of the DNAm signal identified in this study. Overall, the CpGs within MR-ex1 showed generally low DNAm in controls, with methylation levels ranging from 7–24% in males (Fig. 3b) and 3–28% in female macaques (Fig. 3c). In males, LD monkeys showed similar DNAm levels as controls; however, HD/VHD monkeys had increased methylation rates as compared to both controls and LD monkeys (Fig. 3b). In particular, nine CpGs had significantly higher DNAm rates in male HD/VHD monkeys compared with control monkeys. In females, LD subjects could not be included in the analysis due to the small sample size (only 3 subjects). Nonetheless, and similar to heavy ethanol-drinking males, there were four CpGs with significantly higher DNAm rates in the MR-ex1 region of VHD female macaques as compared to control females (Fig. 3c).

Comparison of the MR-ex1 region to human ENCODE data^47^ on 25 chromatin states for seven different brain areas predicted that this region coincides with promoter function (**Supplemental Fig. 3**). Furthermore, and in agreement with a potential role of this region as a promoter^48^, several transcription factors relevant to neuronal regulation and known to have a role in mediating the effects of ethanol on gene regulation are predicted to bind to it, including GR (glucocorticoid receptor^49^), ER-α (estrogen receptor^50^), CREM (cAMP responsive element modulator^51^), CREB (cAMP responsive element binding protein^52^), Sp1^53^, GATA-3^54^, NeuroD1^55^, C/EBP (CCAAT-enhancer-binding proteins^56^) and AP-2α^57^ (**Supplemental Fig. 4**, TRANSFAC^58^). In addition to the fact that this differentially methylated region is located in exon 1 (605 bp downstream of the transcription start site of exon 1A), it is upstream of exons 1B and 1C (~ 28kb and ~ 2kb; respectively), and could act as a regulatory region contributing to differential expression of *KCNN3* transcripts.

We next investigated the DNAm profile of the MR-ex1 region in mice that were drinking ethanol for 22 h in the CIE dependence model. Similar to ethanol-naïve rhesus macaques, DNAm in this region was relatively low in air-exposed control mice. *Kcnn3* methylation levels ranged from 0.1–17%, with average methylation rates for 19 out of 21 CpGs below 5% in the controls. Interestingly, three CpGs showed significantly higher methylation rates in CIE-exposed drinking mice as compared to rates in controls (Fig. 3d). These CpGs are conserved with rhesus macaques and humans (**Supplemental Fig. 2**) and two were located in the binding sites for AP-2α (**Supplemental Fig. 4**).

### Expression of KCNN3 transcript variants differs with ethanol intake levels

We next evaluated the potential relevance of MR-ex1 hypermethylation in regulating *KCNN3* mRNA expression. The mouse (GRCm38.p3) and rhesus macaque (MacaM or Mmul10) genomes are not annotated with as much detail as the human. Thus, in order to investigate the effects of ethanol drinking on the expression of the different *KCNN3* transcripts, we designed primers to amplify two exons common to all reported transcripts (*SK3_ex7/8*), as well as primers to specifically amplify transcript *SK3_ex4, SK3_ex1B*, and *SK3_ex1C* in human. Next, each amplicon’s orthologous sequence was identified in mouse and rhesus macaque, and species-specific primers for the different transcripts were designed. It should be noted that transcripts *SK3_ex1B* and *SK3_ex4* encode dominant-negative and apamin-insensitive isoforms of K_Ca_2.3 channels, respectively, and transcript *SK3_ex1C* was not detected in the NAcC, in agreement with previous studies indicating this transcript is not expressed in the brain^59^.

**Figure 4:**
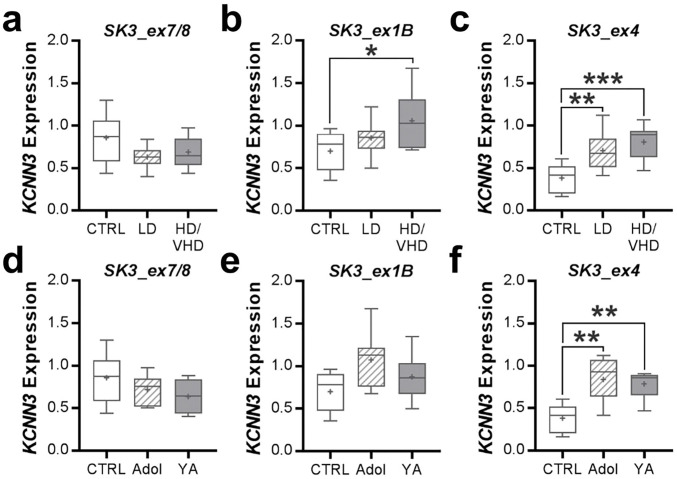
Summary of nucleus accumbens *KCNN3* transcript expression in ethanol drinking rhesus macaques and C57BL/6J mice. **a-c** The relative expression of brain *KCNN3* transcripts (*SK3_ex7/8*, 1 *SK3_ex1B*, and *SK3_ex4*) among the three drinking macaque groups (*SK3_ex1B*: *p = 0.0381 vs CTRL; *SK3_ex4*: **p = 0.0084 vs CTRL, ***p= 0.0009 vs CTRL). **d-f** The relative expression of the *KCNN3* transcripts in drinking monkeys collapsed by age at onset of ethanol drinking (*SK3_ex4*: **p < 0.0015 vs CTRL). **g-i**The relative expression of the *KCNN3* transcript variants in the nucleus accumbens of male C57BL/6J mice that were treated with CIE exposure (*SK3_ex1B*: *p < 0.026 CIE exposed drinkers vs all remaining groups; *SK3_ex4*: **p < 0.015 drinkers vs non-drinkers).

The expression of the exons common to all transcripts (i.e., *SK3_ex7/8*) showed no differences among the different ethanol-drinking male monkey groups (one-way ANOVA: *F*(2, 23) = 2.721, *p* = 0.0870, *n* = 8–9/group; Fig. 4a). However, male monkeys with a HD/VHD phenotype showed a significant increase in expression of transcript *SK3_ex1B* (one-way ANOVA: *F*(2, 22) = 3.547, *p* = 0.0462, *n* = 7–10/group) and *SK3_ex4* (one-way ANOVA: *F*(2, 22) = 9.89, *p* = 0.0009, n = 8–9/group; Fig. 4b,c; **Supplemental Table 4**). In addition, transcript *SK3_ex4* was significantly increased in the accumbens of LD male monkeys (Fig. 4c). Since the male monkeys differed in their age of drinking onset, we wanted to determine if age is an important factor in ethanol regulation of *KCNN3* transcripts levels. Because of the lack of mature adult control monkeys, we could only include samples from adolescent and young adult male monkeys. In controls, expression of the transcripts was not significantly different (*SK3_ex7/8*: two-tailed *t*-test; *t*(2) = 1.802, *p* = 0.2134; *SK3_ex1B*: two-tailed *t*-test; *t*(2) = 0.1494, *p* = 0.8950; *SK3_ex4*: two-tailed *t*-test; *t*(2) = 0.6137, *p* = 0.6019); thus, samples from these two age groups were pooled for further analysis. When collapsed across age, 12 months of open access to ethanol regardless of drinking phenotype significantly increased *KCNN3* transcript *SK3_ex4* expression (one-way ANOVA: *F*(2, 17) = 10.88, *p* = 0.0009; Fig. 4f), but not expression of the two other transcripts (*SK3_ex7/8*: one-way ANOVA: *F*(2, 18) = 1.798, *p* = 0.1941; *SK3_ex1B*: one-way ANOVA: *F*(2, 17) = 2.935; *p* = 0.0803; Fig. 4d,e), in adolescent and young adult male monkeys.

**Figure 5:**
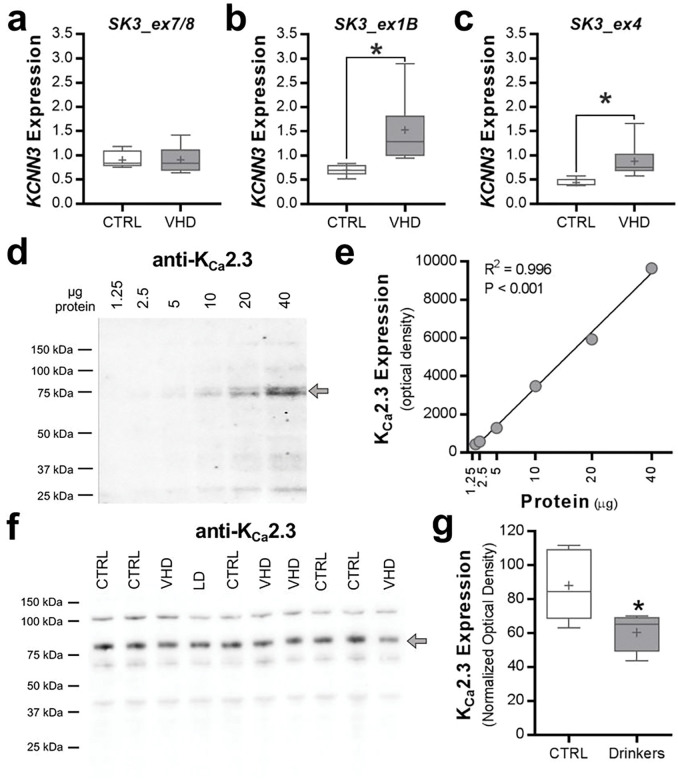
Adaptations in nucleus accumbens *KCNN3* transcript and protein expression in ethanol drinking female rhesus macaques. **a-c** The relative expression of *KCNN3* transcripts (*SK3_ex7/8, 1 SK3_ex1B*, and *SK3_ex4*) among control and very heavy drinking macaque groups (*SK3_ex1B*: *p = 0.037 vs CTRL; *SK3_ex4*: *p = 0.024 vs CTRL). **d** Characterization of anti-K_Ca_2.3 channel western blot in macaque nucleus accumbens tissue (protein loading range, 1.25 – 40 μg). **e** Positive correlation between the amount of protein loaded and anti-K_Ca_2.3 channel optical density values. **f,g** The full K_Ca_2.3 channel blot and quantitation of normalized K_Ca_2.3 channel protein expression in controls and drinkers (*p = 0.0324 vs CTRL).

Because *KCNN3* is hypermethylated in HD/VHD monkeys and transcriptionally regulated by estrogen^60^, analysis of *KCNN3* transcripts was also performed in control and ethanol-drinking female monkeys. Similar to the male monkeys, *KCNN3* transcript *SK3_ex7/8* expression did not differ between the control and VHD female monkeys (two-tailed *t*-test: *t*(8) = 0.036, *p* = 0.972, *n* = 4–6/group; Fig. 5a). Female monkeys with a VHD phenotype showed a significant increase in expression of transcript *SK3_ex1B* (two-tailed *t*-test: *t*(10) = 2.658, *p* = 0.024, *n* = 5–7/group; Fig. 5b) and *SK3_ex4* (two-tailed *t*-test: *t*(9) = 2.477, *p* = 0.037, *n* = 5–6/group; Fig. 5c). All females were of similar age (i.e., 4–6 years old at the start of induction), and no age effect analysis on gene expression was performed. To determine if hypermethylation of *KCNN3* and the shifts in transcript expression affected K_Ca_2.3 channel protein expression, we performed western blot analysis in accumbal tissue from the same female rhesus macaques. Characterization of K_Ca_2.3 channel immunoreactivity in ethanol-naïve monkey NAcC samples revealed a linear dynamic range across twofold dilutions between 1.25 and 40 μg of protein (R^2^ = 0.9963; Fig. 5d,e). Consistent with results from rodent ethanol studies^15, 17, 22^, expression of K_Ca_2.3 channel protein was significantly reduced in VHD female monkeys compared with controls (two-tailed *t*-test: *t(*8) = 2.585, *p* = 0.0324, *n* = 5/group; Fig. 5f,g). Unfortunately, our attempts to detect the different isoforms of K_Ca_2.3 channel, such as the dominant-negative isoform 3 with a predicted molecular weight of 47 kDa, were unsuccessful with commercially available antibodies.

**Figure 6:**
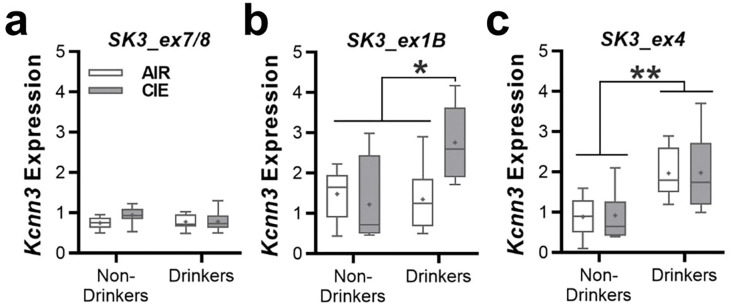
Nucleus accumbens *KCNN3* transcript expression in ethanol drinking and dependent C57BL/6J mice. The relative expression of the *KCNN3* transcript variants **a** *SK3_ex7/8*, **b** *SK3_ex1B*, and **c** *SK3_ex4*in the nucleus accumbens of male C57BL/6J mice that were drinking and were treated with air or CIE exposure (*SK3_ex1B*: *p < 0.026 CIE exposed drinkers vs all remaining groups; *SK3_ex4*: **p < 0.015 drinkers vs non-drinkers).

Given that chronic ethanol drinking and dependence reduced K_Ca_2 currents and expression in the nucleus accumbens of rats and mice^15, 17, 22^, we next determined if *Kcnn3* transcript expression is altered in ethanol dependent mice. Similar to the monkey data, ethanol drinking and/or dependence did not affect Kcnn3 expression of *SK3_ex7/8* (two-way ANOVA: main effect: *F*(1, 50) = 3.931, *p* = 0.0529; Fig. 6a). As with the monkey, dependent mice with access to ethanol in their home cage showed elevated *SK3_ex1B* expression (two-way ANOVA: interaction: *F*(1, 31) = 7.228, *p* = 0.0114; Fig. 6b). Expression of *SK3_ex4* was significantly increased in drinking mice regardless of their history of CIE exposure (two-way ANOVA: *F*(1, 44) = 32.29, p < 0.0001; Fig. 6c).

## Discussion

The results from these studies provide cross-species evidence for alcohol-associated alterations in DNAm signals mapping to *KCNN3* and changes in gene expression in heavy drinking macaques and ethanol dependent mice. In both monkeys and mice, ethanol drinking and dependence was associated with hypermethylation of conserved CpGs at a predicted regulatory region in exon 1A of *KCNN3*. In parallel with the hypermethylation, excessive drinking increased expression of a dominant-negative transcript of *KCNN3* that is transcribed using an alternative exon downstream of exon 1A. Consistent with chronic ethanol-induced loss of apamin-sensitive currents in accumbens and orbitofrontal neurons^15, 17, 61^, ethanol drinking increased expression of the transcript that encodes apamin-insensitive K_Ca_2 channels. We also found a reduction in K_Ca_2.3 channel protein in heavy drinking female macaques that is congruent with decreased expression reported in rodent models of chronic ethanol exposure. These results suggest that ethanol-induced regulation of *KCNN3* transcripts is a conserved mechanism that underlies functional changes in K_Ca_2 channels reported in rodent models.

In the current study, we identified a DMR that maps to an ion channel gene previously implicated in excessive drinking, ethanol-seeking behaviors, and ethanol-induced plasticity of intrinsic excitability^10, 14, 15, 17, 19, 22, 62^. Our analysis identified a DMR that spans exon 1 and part of intron 1 of monkey and mouse *KCNN3* in a region containing CpGs that are highly conserved across species. Across male and female monkeys, more than half of the CpGs in this DMR were hypermethylated in HD/VHD, but not in LD or ethanol-naïve monkeys, an effect that is also conserved in ethanol dependent mice that show escalated drinking. Importantly, the DMR in exon 1 of *KCNN3* coincides with a predicted promoter region with binding sites for neural relevant transcription factors. Furthermore, this DMR is upstream of two alternative exons 1 (1B and 1C). Together, these findings suggest that the DMR is strategically located to potentially regulate alternative transcript expression of *KCNN3*. A previous characterization of the promoter region upstream of the exon 1A transcription start site (TSS) identified consensus sequence binding sites for CREB, AP-1, and AP-2^48^. Additional binding sites for these same transcription factors are predicted to bind to MR-ex1, specifically to significantly differentially methylated CpGs. Numerous signaling transduction pathways that are altered by ethanol consumption lead to CREB activation^63^, a key mediator in the development of addiction. Upon activation, CREB can exert its influence upon target gene transcription and interact with promoter-bound cofactors. Previous evidence showed that CREB modulates BK channel expression^64^, and our results suggest that it may also modulate *KCNN3* expression. AP-1 complexes containing FosB, which accumulates in the NAc after drug intake, modulates promoters of genes relevant to addiction, such as GluA2 and dynorphin^65^. The MR-ex1 region also contains binding sites for glucocorticoids, which have been extensively associated with AUD^66, 67^. Others have shown that glucocorticoids and stress exert profound effects on intrinsic excitability in neurons through regulation of ion channel activity, including K_Ca_2 channels^68–71^. Thus, it is possible that ethanol, by modulating transcription factor levels as well as availability of binding sites by DNAm, modulates *KCNN3* expression.

Using a genome-wide approach, we previously reported DMRs associated with the modulation of genes that regulate synaptic plasticity in the NAcC of heavy drinking monkeys^4^. In a recent whole-genome analysis of the NAcC methylome in additional ethanol-naïve and HD/VHD macaques^5^, several *KCNN3* DMCs (CpG 129,130,076; 129,130,376; 129,130,501; and 129,130,770; Fig. 3B) described in this study reached a nominal p value < 0.05. Interestingly, our recent genome-wide DNAm analysis data of the dorsolateral prefrontal cortex (area A46) collected from alcohol-naïve monkeys revealed that the DNAm rate of several of the differentially MRs included in this study (CpG 129,130,376; 129,130,438; and 129,130,501; Fig. 3B) showed a significant association (p_Sidak_ < 6.36E-05) with alcohol intake levels following prolonged alcohol drinking and repeated cycles of abstinence and relapse (unpublished data). These results suggest that pre-existing DNAm signatures of *KCNN3* and other genes could be a risk factor for future heavy alcohol drinking. In mice, induction of ethanol dependence increased evoked firing in NAcC MSNs^17^ and induced synaptic proteome adaptations in the NAcC^72^. Because alterations in neuronal firing underlie synaptic integration, learning processes and may facilitate drug-associated synaptic remodeling^73, 74^, our findings suggest that a change in the methylation status of key CpGs is a critical cross-species mechanism that might regulate coordinated neuronal excitability and synaptic adaptations that lead to uncontrolled drinking.

In monkeys and mice, heavy ethanol drinking and dependence was associated with increased expression of *KCNN3* transcripts that encode K_Ca_2.3 channels that reduce surface trafficking and apamin sensitivity. A combination of transcripts and protein expression data suggest that with heavy ethanol consumption there is a decrease in transcript *SK3_ex1A* expression while there is an upregulation in transcripts *SK3_ex1B* and *SK3_ex4*. While expression of the dominant-negative transcript was only elevated in heavy drinking monkey, and expression of the apamin-insensitive transcript was increased by ethanol intake regardless of the drinking phenotype or the age of drinking onset. These results are in agreement with our functional and behavioral data on reduced apamin sensitivity in the nucleus accumbens and orbitofrontal cortex of ethanol dependent mice and self-administering rats^15, 17, 61^. We previously reported that apamin microinfusion into the NAcC increased drinking and bath application of apamin reduced K_Ca_2-mediated currents in NAcC MSNs in non-dependent C57BL/6J mice. However, the ability of apamin to influence drinking and K_Ca_2 currents was completely lost when mice were exposed to CIE. Moreover, ethanol intake and evoked firing in NAcC MSNs were increased and there was a reduction in K_Ca_2 channel currents and protein levels in the ethanol dependent mice. Although it is unknown if heavy ethanol drinking in macaques alters firing properties of NAcC MSNs, the shift in transcripts and the reduction in total K_Ca_2 channel protein suggests that prolonged ethanol intake increases intrinsic excitability similar to results from rodent models of ethanol self-administration^15^ and dependence^17, 75^. In addition to reduced Kcnn3 gene expression and increased intrinsic excitability, a recent study reported a loss of apamin’s ability to increase evoked firing in cultured cortical neurons treated with DNA methyltransferase inhibitors^37^. Thus, these data provide support that the increase in *SK3_ex1B* and *SK3_ex4* transcript expression through hypermethylation of *KCNN3* exon 1A is an underlying mechanism driving these functional and behavioral adaptations across species and brain regions.

Previous studies using functional genomics and rodents with divergent drinking phenotypes have identified *Kcnn3* as a candidate signature gene that is associated with binge-like and excessive ethanol drinking^10, 13, 17–19, 21^. In contrast to mounting evidence and our hypothesis, high numbers of polymorphic trinucleotide repeats encoded by exon 1 of *KCNN3* did not segregate with a heavy drinking phenotype in this population of rhesus macaques. There are a number of possibilities that could explain these negative findings. Although long CAG repeats in K_Ca_2.3 channels reduced apamin-sensitive currents^24^, function of this polymorphism was characterized in transfected HEK293 cells and not mammalian central neurons. In the current study, CAG repeat number was measured from blood samples taken after chronic ethanol consumption. While traditionally considered stable, there is some evidence that CAG repeats can vary between types of tissue (i.e, peripheral vs central) and can expand across time to accelerate disease progression^76, 77^. Thus, future longitudinal studies are necessary to track CAG repeat number in the NAcC of LD and HD/VHD monkeys.

In summary, these cross-species findings of genetic and epigenetic adaptations in *KCNN3* by excessive alcohol consumption represent a complex mechanism through the use of alternative promoters that likely impact intrinsic excitability of NAcC MSNs, and, ultimately, ethanol-seeking behaviors. We propose a model in which MR-ex1 functions as a regulatory region to modulate the expression of the alternative transcripts *SK3_ex1B* and *SK3_ex4*. Our findings provide the first evidence that hypermethylation of the MR-ex1 region of *KCNN3* by heavy alcohol drinking is a key cross-species mechanism that may be important for the maintenance of excessive drinking and the development of AUD.
